# Surmounting a PCR challenge using a Contradictory matrix from the Theory of Inventive Problem Solving (TRIZ)

**DOI:** 10.1186/s40064-015-1577-3

**Published:** 2016-01-20

**Authors:** Jiří Drábek

**Affiliations:** Faculty of Medicine and Dentistry, Institute of Molecular and Translational Medicine, Palacký University, Teaching Hospital Olomouc, Hněvotínská 5, 779 00 Olomouc, The Czech Republic

**Keywords:** CADMA PCR, Contradictory matrix, Invention, TRIZ

## Abstract

In this paper I tested whether Contradictory Matrix with 40 Inventive Principles, the simplest instrument from the Theory of Inventive Problem Solving (TRIZ), is a useful approach to a real-life PCR scenario. The PCR challenge consisted of standardization of fluorescence melting curve measurements in Competitive Amplification of Differentially Melting Amplicons (CADMA) PCR for multiple targets. Here I describe my way of using the TRIZ Matrix to generate seven alternative solutions from which I can choose the successful solution, consisting of repeated cycles of amplification and melting in a single PCR run.

**Electronic supplementary material:**

The online version of this article (doi:10.1186/s40064-015-1577-3) contains supplementary material, which is available to authorized users.

## Background

CADMA is a modified form of PCR based on competition between two partially overlapping forward primers for a DNA template (Kristensen et al. 2012a; Borgbo et al. 2014; Kristensen et al. 2012b), one of which binds to both wildtype and mutated templates while the other is mutation-specific. The reverse primer binds to both wildtype and mutated templates. When optimized, CADMA PCR with a heterozygous (wildtype plus mutant) DNA template amplifies two sequences, while wildtype homozygous templates provide only one amplicon. The melting temperature of the heterozygote is distinguishable from that of the homozygote by its upward or downward shift of a melting temperature (T_m_) peak, or by the presence of a second T_m_ peak. When a second T_m_ is present, calculation of the mutant to wildtype ratio is possible (data not shown, to be published elsewhere). However, my colleagues and I have encountered a difficulty in simultaneous CADMA analyses of several targets using multiple DNA samples in a single thermocycler.

**Fig. 1:**
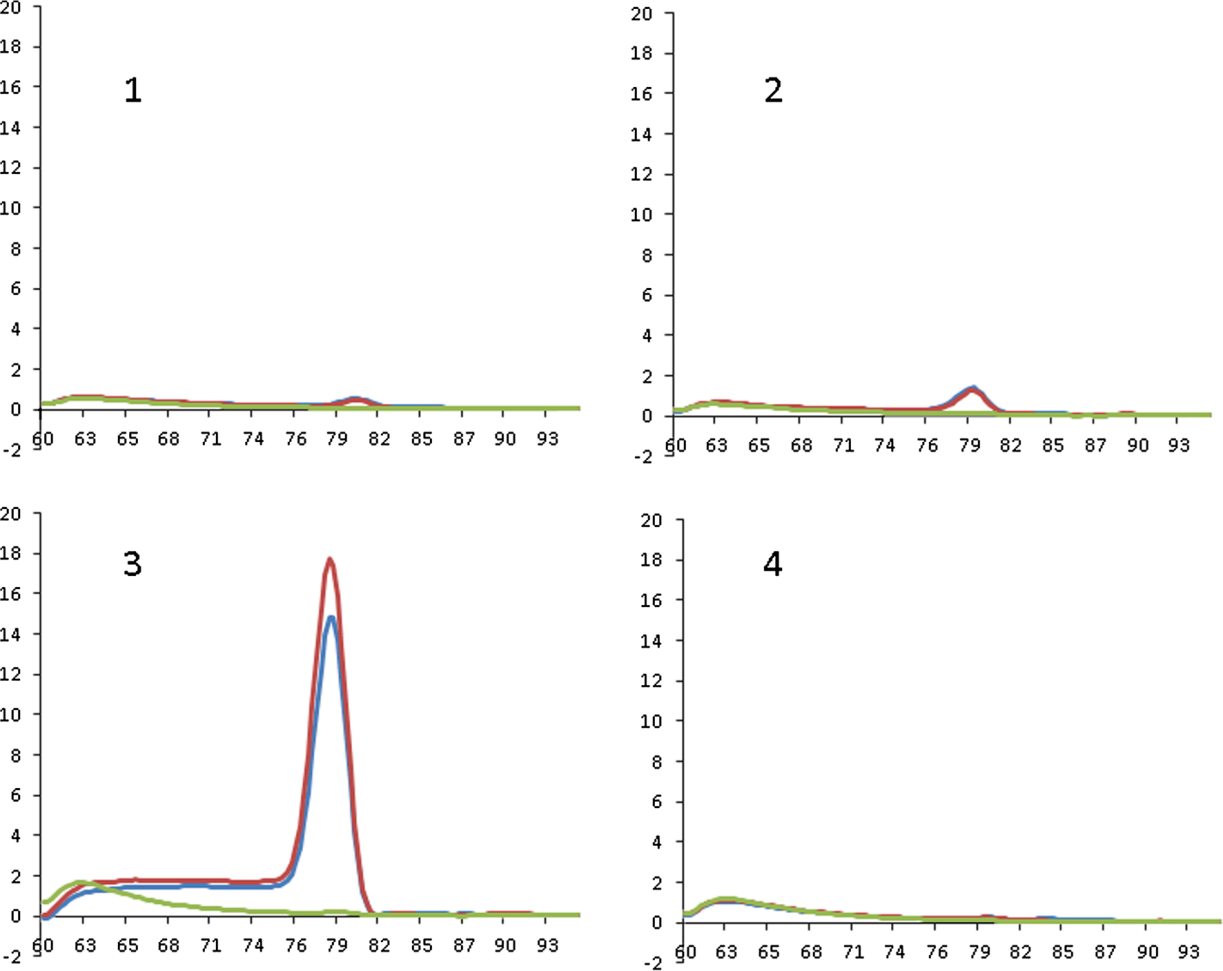
Unoptimal CADMA PCR fluorescence signals after 25 cycles. Axis x corresponds to temperature, axis y to fluorescence. CADMA was performed with a water blank (*green line*) and two samples of wildtype DNA (*blue and red lines*) using primer mixes for codon 59 of *KRAS* (**1**), codon 61 of *KRAS* (**2**), codon 59 of *NRAS* (**3**), and codon 61 of *NRAS* (**4**). Only mixture 3 (for codon 59 of *NRAS*) yielded a satisfactory signal

We have been applying CADMA PCR to the detection of *RAS* mutations in formalin-fixed parafin-embedded samples from colorectal carcinoma patients. These are patients who are to be prescribed anti-EGFR therapy (panitumumab/cetuximab) on the basis of their *RAS* wildtype status (Kasi et al. 2015). Using our general thermocycling parameters, (95 °C 15′, (95 °C 10′′, 66 °C 20′′ touchdown −1 °C per cycle, 72 °C 20′′) × 10, (95 °C 10′′, 58 °C 20′′, 72 °C 20′′) × 25, melt to 95 °C at 10 acquisitions per °C) and primers designed for this purpose (described in Additional file 1), we observed an unequivocal melting signal for the primer mixture for *NRAS* codon 59, while primer mixes for the *KRAS* codon 59 and 61 and for the *NRAS* codon 61 did not yield a sufficient signal (Fig. 1).

**Fig. 2:**
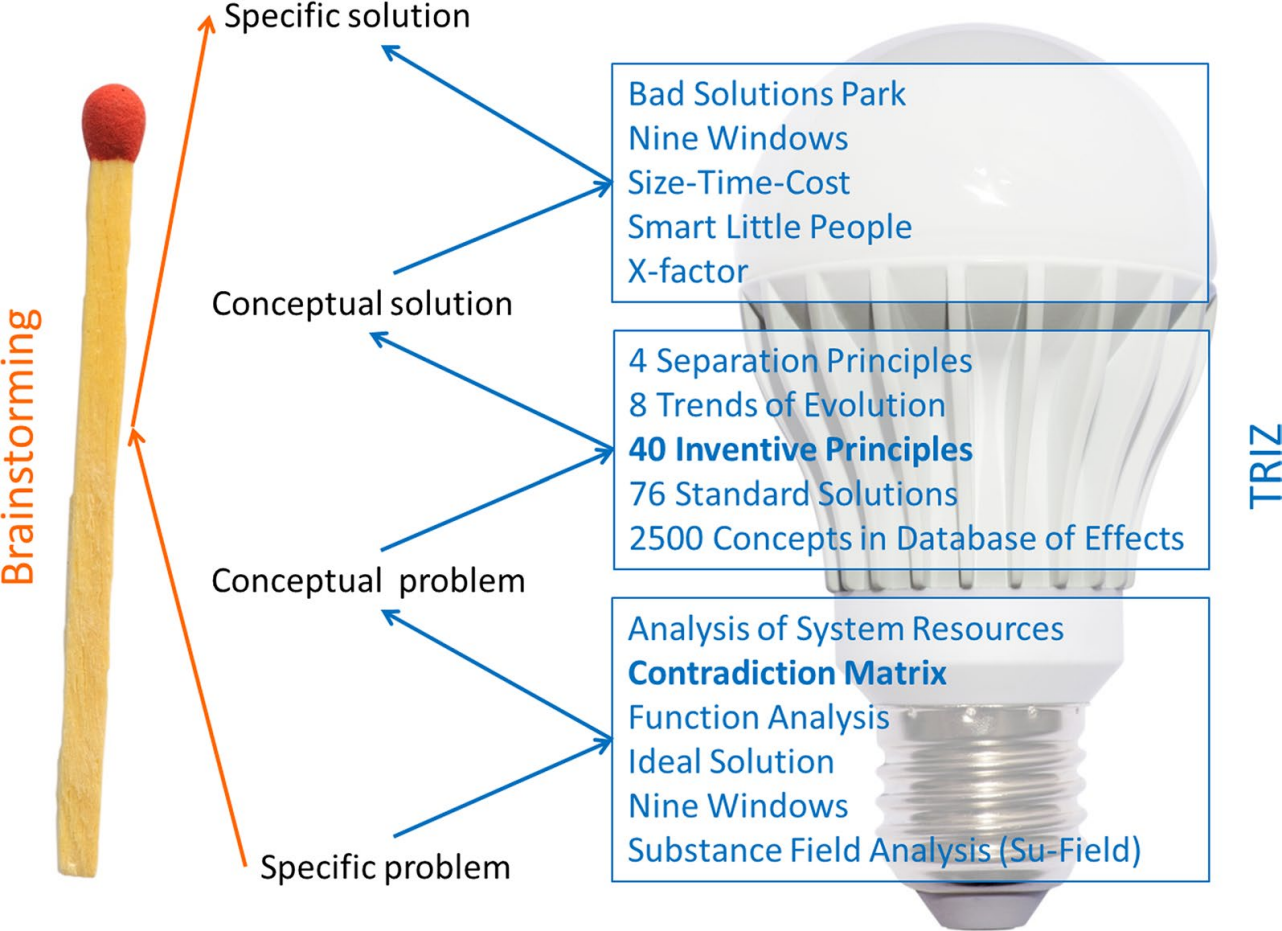
Main components of TRIZ in comparison with brainstorming. The simplest TRIZ methods, used and discussed in this paper, are in bold. For illustration of the broad TRIZ portfolio of solution options, the names of other methods are depicted in the figure. For an explanation of the principles of these methods, readers are advised to consult the textbook of Gadd (2011) or Internet (i.e. http://www.xtriz.com/Annotated%20list%20of%20main%20TRIZ%20tools%20and%20techniques.pdf). Bulb photo ©Depositphotos.com/[Dmitry Raykin] and match photo ©Depositphotos.com/[Sommai Larkjit]

We were unable to achieve satisfactory results by standardizing the quantity of template DNA, by adjusting the concentration of primers/magnesium/Taq polymerase and even PCR annealing temperature and buffers. The PCRs appear to proceed at different rates and to different degrees of completion. Some of the PCRs plateau or accumulate reaction-hindering artefacts (primer dimers, non-specific and concatenated amplicons) by the time other reactions have begun their exponential phase. Because brainstorming did not throw up any solutions, we opted for the more systematic Theory of Inventive Problem Solving (TRIZ, acronym derived from the Russian Teopия Peшeния Изoбpeтaтeльcкиx Зaдaч) (Altshuller 1996). Although innovation is clearly a driving force of technological advancement, to my knowledge, TRIZ has not been systematically applied to address challenges in molecular genetics. The key components of TRIZ (Ilevbare et al. 2013; Moehrle 2005) consist of the formalization and abstraction of problems, the mining of databases of abstract solutions, and concretization of solutions (Fig. 2).

This paper describes a protocol of the simplest TRIZ method, a 39 × 39 Contradiction Matrix with 40 Inventive Principles, applied to the problem of optimization of reactions for multiple targets in CADMA (Competitive Amplification of Differentially Melting Amplicons) PCR.

## Methods

The TRIZ Contradiction Matrix is a two-dimensional table where 39 engineering parameters to be improved (i.e. speed, force, and temperature) are positioned in rows while the same 39 parameters, that can be conflicting, are positioned in columns. The intersection of a row and a column for a given problem of desired and undesired property enlists up to 4 Inventive Principles (i.e. Segmentation, Asymmetry, and Combination) from 40 principles most often encountered in patent databases (Additional file 2).

The application of the Contradiction Matrix was performed by identification of technical contradictions, suggestion of abstract solutions, customizing of the solutions, evaluating the solutions, and testing the chosen solution in the laboratory.

## Results

### Step 1: identification of technical contradictions

In our CADMA challenge, we desire to obtain an unambiguous signal for homozygotic and heterozygotic templates with a similar fluorescence intensity for each of the multiple genetic targets.

The closest or most relevant abstract terms for these phenomena in the Contradiction matrix in my opinion were 27—Reliability (I want to achieve an unambiguous, reliable result of mutation *vs* wildtype) and 18—Brightness (I do not want to lose any fluorescence signal intensity from some of the target amplicons).

### Step 2: suggestion of abstract solution

To address this combination of conflicting properties, the TRIZ matrix (Additional file 2) gives the following hints: Cushion in advance (11), Change the colour (32), and Invert an object, system or process (13). Let us consider if (and how) each of these Principles could be applied to solve the problem.

### Step 3: customizing the solution

First, in accordance with the “Cushion in advance” principle it may be possible to pre-set the optimal number of PCR cycles, by:

1. Standardizing the fluorescence threshold (C_t_) used to characterize amplification.
2. Using a limiting amount of PCR reagents or degradable PCR reagents (Du Breuil and Rusla 2014).
3. Changing the PCR profile to “repetitive melting by design”.

Second, in accordance with the “Changing colour” Principle it may be possible to:

4. Make the instrumentation responsive to the fluorescence colour threshold and automatically record it, or stop the PCR reaction.
5. Introduce modifications that cause the destruction of PCR reagents when products are either amplified or detected (the “colour” changes), thus hindering further amplification.

Third, in accordance with the Inversion Principle, it may be possible to:

6. Broaden the range of appropriate number of cycles by enhancing the specificity of primer binding, rather than attempting to identify the optimum number of cycles.
7. Monitor distinct PCR products in real time instead of distinguishing them after PCR by their melting curves.

### Step 4: evaluating the solution

Let us assess these potential solutions with regard to available resources and our goal.

- *Solution 1* Fluorescence standardization could be achieved by standardizing DNA inputs (using quantitative PCR to quantify DNA samples before genotyping with PCR). Pros: inexpensive as it limits the quantity of reagents required, with no need for non-standard procedures, reagents, and instruments. Cons: time consuming, may not solve the problem completely and will not account for changes in quality of PCR components other than DNA.
- *Solution 2* Limiting reagents or degradable primers could theoretically bring a PCR to the plateau phase before the start of undesired amplification of wildtype template by the mutation-specific primer. Pros: inexpensive, with no need for nonstandard procedures, reagents or instruments. Cons: limiting reagents may not solve the problem (both pre-plateau and plateau states may be primer-dependent), protective chemicals would be needed for degradable primers, and the detection limit may be shifted.
- *Solution 3* Repetitive melting involves changing the procedure from standard PCR, with an adjustable cycling stop and a single melting, to repeated sequences of amplification cycles and melting. Pros: inexpensive, with no need for nonstandard procedures, reagents or instruments. Cons: time consuming even for runs that do not require the modification.
- *Solution 4* A fluorescence threshold valve is potentially elegant but not possible with current thermocyclers. Pros: universal, adaptable solution. Cons: requires the development of instruments or software with new functions.
- *Solution 5* The responsive destruction of reagents is a theoretical one. Pros: it could potentially provide clear signals distinguishing wildtype homozygotes from heterozygotes. Cons: it would require more innovation and optimization to balance the amplification power of the residual PCR reagents with the power of PCR products to degrade the same (PCR) reagents.
- *Solution 6* An increase in specificity could be achieved using locked nucleic acids (Braasch et al. 2001), peptide nucleic acids (Nielsen et al. 1994), or cooperative primers (Satterfield 2014). Pros: it could potentially provide clear signals distinguishing wildtype homozygotes from heterozygotes. Cons: it would require novel reagents and the design of the required primers may not be trivial.
- *Solution 7* A real-time monitoring of the specific target involves radically changing the principle used to detect products (e.g. by using Scorpion primers (Thelwell et al. 2000) or FRET duos of probes (Wang et al. 2010). Pros: avoids the problem of identifying the appropriate number of cycles. Cons: simplicity of CADMA is lost, and special reagents are required.

### Step 5: Testing the solution in the laboratory

**Fig. 3:**
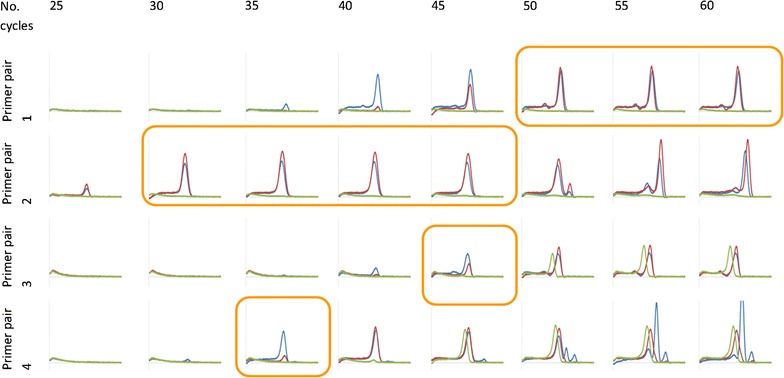
TRIZ solution of CADMA PCR challenge. CADMA was performed with the same primer mixes, templates, and reaction mixtures as in Fig. 2. The interpretation windows (with orange lining) for primer mixes 1–4 are cycles 50–60, 30–45, 45, and 35, respectively. Primer mix 2 generated a concatenated artefact from the 50th cycle, primer mix 3 dimerized from the 45th cycle, while mix 4 dimerized from the 45th cycle and generated concatenated products from the 50th cycle. Though there is no universally suitable number of cycles for the four primer mixes and two DNA samples, TRIZ-derived PCR protocol enables a result for each primer mix after a single PCR run

The simplest and least expensive solutions appear to be both solutions 1 and 3. As shown in Fig. 3, simultaneous application of these solutions solves the problem. For each primer mix, at least one cycle (followed by melting) was found to have satisfactory results. In the event that multiple cycles were satisfactory, the best of them could have been chosen by maximizing the difference in melting temperatures of the wildtype and mutant (data not shown).

## Discussion

I found seven potential solutions to our PCR CADMA problem using the Contradiction Matrix with Inventive Principles. While some solutions are obvious to the informed reader (such as the standardization of DNA input), it may be speculated that TRIZ-guided Inventive Principles provided more focus and a more systematic path to the “repetitive melting by design” solution. A combination of two TRIZ-guided approaches has been successfully tested. The Contradictory Matrix may have been used in another way. For example, the intersection of 12 (Shape of the melting curve) and 28 (Accuracy of measurement of mutated DNA) provides three solutions: 28 (Replacement of mechanical system), 32 (Changing the colour), and 1 (Segmentation). However, solution 32 was suggested even by the original contradictory combination of 27 (Reliability) and 18 (Brightness). The Segmentation principle of solution 1 may be applied to repetitive melting segments within one PCR run, thus yielding the same tangible solution. It remains to be seen if it is necessary to choose the best contradictory terms or whether the TRIZ process of abstraction and concretization confers more benefits than does brainstorming.

Although this solution, or a more elegant one, may have been identified by innovative scientists even without the Contradictory Matrix, the TRIZ approach enabled a better solution to our problem than did brainstorming.

It may be argued that this application of TRIZ was anecdotal and that the Contradictory Matrix is not suitable for bioanalysis with preanalytical and analytical requirements substantially differing from the 39 original engineering parameters. Nevertheless, my application of TRIZ in molecular genetics was successful and warrants further testing. It is possible that a new Altshuller-like datamining approach to solutions of genetic diagnostics problems would provide a different and more apt Contradiction Matrix. This endeavour was behind the scope of this article. Big data approaches together with crowdsourcing seem amenable to this task.

## Conclusion

In this paper I tested the simplest TRIZ instrument, the Contradictory Matrix with 40 Inventive Principles, for use in genetic diagnostics. I chose a real-life scenario for this purpose. My challenge was to determine the optimal conditions in Competitive Amplification of Differentially Melting Amplicons Polymerase Chain Reaction (CADMA PCR) and I found a functional solution using the Contradictory Matrix.

Testing or meta-testing the TRIZ on a representative number of molecular genetics challenges may be required to fully assess its potential. It is suggested that the Contradictory Matrix terminology and knowledge base be modified and broadened for successful application in a new non-technical domain.

## Additional files

10.1186/s40064-015-1577-3 Minimum Information for Publication of Quantitative Real-Time PCR Experiments (MIQE) Information for CADMA PCR solution.
10.1186/s40064-015-1577-3 Altshuller´s Contradiction Matrix and Forty Inventive Principles. Upon entering desired parameter (in green) and undesired property (in red), suggested solution appears with yellow background.
