# Supplementary material for: Surmounting a PCR challenge using a Contradictory matrix from the Theory of Inventive Problem Solving (TRIZ)
Source: Springerplus. 2016 Jan 20;5:56. doi: 10.1186/s40064-015-1577-3 (PMC4720617; doi:10.1186/s40064-015-1577-3)
Supplement: Supplementary file 1 — 10.1186/s40064-015-1577-3 Minimum Information for Publication of Quantitative Real-Time PCR Experiments (MIQE) Information for CADMA PCR solution. [file 40064_2015_1577_MOESM1_ESM.doc]

## MIQE Information for PCR

Experiment title: *RAS* CADMA; Performed by: Miroslava Rabčanová, Veronika Holinková, Eva Hrušková; Date: 25.6.2014 – 25.9.2014; Institution: IMTM; Sample Preparation & Nucleic Acid Isolation

Material: Human colorectal tumour FFPE sample, three microtome slices 10 µm each and human blood sample, 5 ml in EDTA; Study set up: DNA *RAS* wildtype from blood, DNA *RAS* wildtype from FFPE, and water with 12 primer mixes each. Primer mixes aimed to detect mutations: *KRAS* 59T, *KRAS* 61 *NRAS* 59, and *NRAS* 61RLK. Handling: FFPE sample was sliced with microtome (3 x 5 µm) before DNA extraction. DNA was extracted from the blood sample on the same day day that the blood was drawn. ; Method of processing and preservation: FFPE sample was one month old from time of biopsy; EDTA blood sample was kept at room temperature; Extraction method:

DNA was extracted from FFPE using the cobas DNA kit without modification of the protocol, using supplied elution buffer; Blood DNA was extracted using MC101 kit on MagCore without modification of the protocol, using the supplied elution buffer. RNase was not used. Nucleic acid purity and integrity was checked using Nanodrop ND1000.

FFPE sample: c = 80 ng/µl, A_260_/A_280_ = 1.88, V = 50 µl. Blood sample: c = 145, A_260_/A_280_ = 1.76, V = 100 µl. We have not checked for presence of PCR inhibitors because none was expected; PCR Amplification and Analysis: Target sequence for *KRAS* 59T, *KRAS* 61 primers >grch37:12:25357723:25403870:1

TCCCTTCTCAggATTCCTACAggAAgCAAgTAgTAATTgATggAgAAACCTgTCTCTTggATATTCTCgACACAgCAggTCAAgAggAgTACAgTgCAATgAgggACCAgTACATgAggACTggggAgggCTTTCTTTgTgTATTTgCCATAAATAATACTAAATCATTTgAAgATATTCACCATTATAggTgggTTTAAATTgAATATAATAAgCTgACATTAAggAgTAAT

Target sequence for *NRAS* 59, *NRAS* 61RLK primers

>gi|166706796|gb|EU332857.1| Homo sapiens neuroblastoma RAS viral (v-ras) oncogene homolog (NRAS) gene, complete cds

ATgCTTATTTAACCTTggCAATAgCATTgTATTCCCTgTggTTTTTAATAAAAATTgAACTTCCCTCCCTCCCTgCCCCCTTACCCTCCACACCCCCAggATTCTTACAgAAAACAAgTggTTATAgATggTgAAACCTgTTTgTTggACATACTggATACAgCTggACAAgAAgAgTACAgTgCCATgAgAgACCAATACATgAggACAggCgAAggCTTCCTCTgTgTATTTgCCATCAATAATAgCAAgTCATTTgCggATATTAACCTCTACAggTACTAggA

*In silico* specificity screen for wildtype primers was performed using BLAT (<https://genome.ucsc.edu/cgi-bin/hgPcr?wp_target=&db=hg38&org=Human&wp_f=&wp_r=&wp_size=4000&wp_perfect=15&wp_good=15&wp_showPage=true&hgsid=389447661_gokjX3638gkqZaudPaTrNmfjrSB2>): Forward wt; Reverse wt; BLAT PCR result

*KRAS* A59T, gAgAAACCTgTCTCTTggATA, ATTgCACTgTACTCCTCT >chr12:25227324-25227381; *KRAS* Q61R, CCCTCATTgCACTgTACTCCTC, TTggATATTCTCgACACAgCA >chr12:25227319+25227367; *NRAS* A59TD; gAAACCTgTTTgTTggACATA, ATggCACTgTACTCTTCTTgT >chr1:114713890-114713945; *NRAS* Q61RLK, TTggACATACTggATACAgCT, gTCTCTCATggCACTgTACTCTTC >chr1:114713883-114713933; Secondary structure analysis of amplicon was performed using uMelt (<https://www.dna.utah.edu/hets/umh.php>);

**Table 1. Primer sequences, concentrations, and amplicon lengths.**

|  | **Forward** | **µM** | **Reverse** | **µM** | **Amplicon length (bp)** |
| --- | --- | --- | --- | --- | --- |
| ***KRAS* A59T** | *gAgAAACCTgTCTCTTggATA* | 0.1 | *CATTgCACTgTACTCCTCT* | 0.1 | 58 |
|  | *CCTgTCTCTTggcTATTCTCgcCACAA* | 0.4 |  |  | 52 |
| ***KRAS* Q61R** | *CCCTCATTgCACTgTACTCCTC* | 0.1 | *TTggATATTCTCgACACAgCA* | 0.1 | 49 |
|  | *CTTggAcATTCTCgACACAgCAcgTCg* | 0.4 |  |  | 51 |
| ***NRAS* A59TD** | *gAAACCTgTTTgTTggACATA* | 0.05 | *ATggCACTgTACTCTTCTTgT* | 0.05 | 56 |
|  | *CCTgTTTgTTcgACATACTcgATACAA* | 0.2 |  |  | 52 |
|  | *CTaTTTgTTggACATcCTggATACAgA* | 0.2 |  |  | 51 |
| ***NRAS* Q61RLK** | *TTggACATACTggATACAgCT* | 0.1 | *gTCTCTCATggCACTgTACTCTTC* | 0.1 | 51 |
|  | *gTcggACATACTggATAaAgCTggACg* | 0.4 |  |  | 52 |
|  | *TgTTggACcTACTggATACAgaTggAA* | 0.4 |  |  | 53 |
|  | *gTTagACATACTggATcCAgCTggACT* | 0.4 |  |  | 52 |

Manufacturer of oligonucleotides: Generi Biotech, the Czech Republic; Oligo purification method: standard; qPCR reaction volume: 10 µl; Amount of DNA per PCR reaction: 10 ng; Buffer reagents concentrations: PCR buffer (1*), MgCl_2_ (1.5 mM), dNTPs (0.2 mM), ThermoTaq (0.05 U/µl), EVAgreen (1*), primers (see Table above); Polymerase identity: Thermo Scientific AB0908, 10* PCR buffer AB-1217; Plastics: white Roche reaction plates; Final thermocycling parameters: 95°C 15´, (95°C 10´´, 66°C 20´´ touchdown -1°C per cycle, 72°C 20´´)*10, (95°C 10´´, 58°C 20´´, 72°C 20´´)*25, 95°C 1´, 60°C 45´´, melt to 95°C at 10 acquisition per °C, (95°C 10´´, 58°C 20´´, 72°C 20´´)*5, 95°C 1´, 60°C 45´´, melt to 95°C at 10 acquisition per °C, (95°C 10´´, 58°C 20´´, 72°C 20´´)*5, 95°C 1´, 60°C 45´´, melt to 95°C at 10 acquisition per °C, (95°C 10´´, 58°C 20´´, 72°C 20´´)*5, 95°C 1´, 60°C 45´´, melt to 95°C at 10 acquisition per °C, (95°C 10´´, 58°C 20´´, 72°C 20´´)*5, 95°C 1´, 60°C 45´´, melt to 95°C at 10 acquisition per °C, (95°C 10´´, 58°C 20´´, 72°C 20´´)*5, 95°C 1´, 60°C 45´´, melt to 95°C at 10 acquisition per °C, (95°C 10´´, 58°C 20´´, 72°C 20´´)*5, 95°C 1´, 60°C 45´´, melt to 95°C at 10 acquisition per °C, (95°C 10´´, 58°C 20´´, 72°C 20´´)*5, 95°C 1´, 60°C 45´´, melt to 95°C at 10 acquisition per °C; Reaction setup: manual; Equipment: Roche LC480 II; Cq of the non-template control reaction: Cycle number *KRAS* A59T >60, *KRAS* Q61R >60, *NRAS* A59TD 50, *NRAS* Q61RLK 45; PCR efficiency was not calculated because no quantification was intended; Positive control DNA was not used because the primer mixes are not yet sufficiently optimized to be used for testing the *RAS* status of the FFPE samples. Water (no template control, NTC) was used to check for primer dimers (that appeared in *NRAS* A59TD and *NRAS* Q61RLK reactions); Data analysis: Roche software Release 1.5.0 SP4 supplied with cycler was used to analyze the data. Raw fluorescence melting data were exported to MS Excel where fluorescence curve was made more visible by thickening the line; Experiment was repeated three times with similar results and the same conclusion regarding the correct number of cycles.
